# Disease-specific Mortality of Differentiated Thyroid Cancer With Distant Metastases

**DOI:** 10.1210/jendso/bvaf034

**Published:** 2025-02-24

**Authors:** Ali Howaidi, Anwar Alswailem, Abdulrhman Hakami, Afnan Hadadi, Deema Alturki, Fayha Abothenain, Lulu Alobaid, Najla Saleh Ewain, Avaniyapuram Kannan Murugan, Ali S Alzahrani

**Affiliations:** Department of Pathology and Laboratory Medicine, King Faisal Specialist Hospital and Research Centre, Riyadh 11211, Saudi Arabia; Department of Pathology, King Fahad Medical City, Riyadh 11525, Saudi Arabia; Department of Pathology and Laboratory Medicine, King Faisal Specialist Hospital and Research Centre, Riyadh 11211, Saudi Arabia; Department of Medicine, King Faisal Specialist Hospital and Research Centre, Riyadh 11211, Saudi Arabia; Department of Medicine, King Faisal Specialist Hospital and Research Centre, Riyadh 11211, Saudi Arabia; Department of Medicine, King Faisal Specialist Hospital and Research Centre, Riyadh 11211, Saudi Arabia; Department of Medicine, King Faisal Specialist Hospital and Research Centre, Riyadh 11211, Saudi Arabia; Department of Medicine, King Faisal Specialist Hospital and Research Centre, Riyadh 11211, Saudi Arabia; Department of Medicine, King Faisal Specialist Hospital and Research Centre, Riyadh 11211, Saudi Arabia; Department of Medicine, King Abdulaziz Medical City, Riyadh 11426, Saudi Arabia; Department of Molecular Oncology, King Faisal Specialist Hospital and Research Centre, Riyadh 11211, Saudi Arabia; Department of Medicine, King Faisal Specialist Hospital and Research Centre, Riyadh 11211, Saudi Arabia; Department of Molecular Oncology, King Faisal Specialist Hospital and Research Centre, Riyadh 11211, Saudi Arabia

**Keywords:** thyroid cancer, differentiated thyroid cancer, distant metastases, mortality, lung metastases, bone metastases

## Abstract

**Overview:**

Distant metastases (DM) are the major cause of death in patients with differentiated thyroid cancer (DTC). This study aimed to investigate the predictors of DM-associated mortality.

**Patients and Methods:**

We identified 154 thyroid cancer (TC) patients with DM from our institution's tumor registry. We excluded anaplastic (n = 21) and medullary TC (n = 32) and patients with inadequate data (n = 15). The remaining 86 patients with DTC were studied. These include 57 females (66.3%) and 29 males (33.7%) with a median age of 53.5 years [interquartile range (IQR) 45-65]. All patients underwent thyroidectomy; 58 (67.4%) had neck dissection, and 81 (94.2%) received radioactive iodine (I-131) ablation/therapy.

**Results:**

Lung metastases were the most common, occurring in 91.9%; skeletal metastases occurred in 58.1%, brain metastases in 9.3%, and multiple-organ DM in 58%. The management of DM included surgery, 1 or more doses of I-131, external beam radiotherapy, and multikinase inhibitors. Over a median follow-up of 84 months (IQR 35.5-118) for the whole cohort, 47 patients succumbed to their disease (disease-specific mortality 54.7%). Factors associated with mortality were increasing age (*P* = .001) and bone metastases (*P* < .0001). These factors remained significant in multivariate analyses [for age, *P* = .009, hazard ratio (HR) 1.030, 95% confidence interval (CI) 1.007-1.053] and for bone metastases (*P* = .017, HR 2.58, 95% CI 1.19-5.6).

**Conclusion:**

DM from DTC are associated with ∼ 55% mortality at a median survival of 47 months. Increasing age and skeletal metastases are predictors of an increased risk of mortality.

The incidence of differentiated thyroid cancer (DTC), comprised of papillary, follicular, and oncocytic types, has been increasing over the past few decades [1, 2]. This increase in incidence occurred mostly in papillary thyroid cancer (PTC) and is explained largely by the common use of neck ultrasonography and other imaging studies. These commonly used radiological studies detect small-size DTCs that would not have been detected otherwise and are unlikely to cause morbidity or mortality [3]. The vast majority of these tumors are of low risk with an overall survival of more than 95% [2, 4]. However, in about 10% to 15% of cases, DTC is more aggressive and carries a higher risk of morbidity and mortality [4-6]. In these cases, there is a significant risk of locally invasive disease and the development of distant metastases (DM). Although the risk of DM is low at about 1.2% to 13% in most series [7-12], they are the major cause of death in patients with DTC. DM commonly involve the lungs, skeleton, occasionally the brain, and other organs [7-12]. During the past 15 years, many advances in the management of DTC have taken place, including changes in the approach to surgical therapy and radioactive iodine (I-131) administration, advances in external beam radiotherapy, and the common use of targeted therapy in progressive radioiodine refractory DTC cases [4, 13]. These advances are expected to have an impact on the management and outcome of patients with DM. However, mortality remains significant in patients with DM arising from DTC [5, 13]. Therefore, we aimed to examine the rate and risk factors associated with mortality in patients with DTC DM.

## Patients and Methods

We obtained an institutional review board approval from the Ethics Committee of the King Faisal Specialist Hospital and Research Centre, Riyadh, Saudi Arabia (RAC# 2013006). A waiver of consent was granted since the study was retrospective. We searched our institution's tumor registry between January 1, 2005, and January 1, 2020, for cases of DTC with DM. We limited the search to the beginning of 2020 for adequate follow-up time to assess long-term outcomes. Of 4470 thyroid cancer patients seen during this timeframe, we identified 154 patients (3.4%) with DM from thyroid cancer. We excluded patients with anaplastic (n = 21) and medullary (n = 32) thyroid cancers and cases with short follow-up or inadequate data (n = 15). We excluded anaplastic and medullary thyroid cancers since these tumors are associated with high rates of DM and mortality and would skew data on these outcomes. We wanted to focus on DTC and its subtypes only since these are the most common types of thyroid cancer. After these exclusions, 86 patients who had DTC with DM were studied. We collected data on patient demographics, surgical management, histological classification, tumor size, TNM stage, medical management and outcome.

## Statistical Analysis

Continuous data are expressed as median and range or interquartile range and analyzed using T-test and Wilcoxon rank sum tests. Categorical data are expressed as rates and percentages and analyzed by the χ^2^ or Fisher’s exact tests. A multivariate logistic regression analysis was used to analyze the factors associated with disease-associated mortality. Cox proportional hazards and Kaplan-Meier analyses with log-rank tests were used to analyze survival and factors associated with survival. A 2-tailed *P*-value of < .05 was considered statistically significant.

## Results

### Initial Characteristics and Management

**Figure 1.**
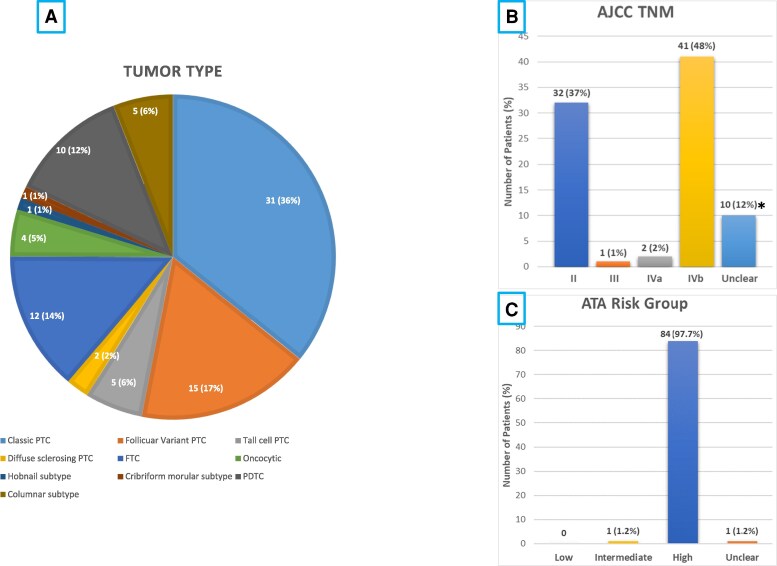
Pie chart showing the distribution of the different subtypes of thyroid cancer in the 86-patient cohort included in this study (A). The AJCC TNM (B) and American Thyroid Association risk groups (C) are also shown. *These patients developed distant metastases at a later stage during their follow-up (metachronous metastases) and information on tumor size, extrathyroidal extension/invasion, and/or location/extent of lymph node metastases at the time of initial surgery was not available.

A total of 86 DTC patients with DM were studied (Table 1). These include 57 females (66.3%) and 29 males (33.7%) with a median age of 53.5 years [interquartile range (IQR) 45-65 years]. Patients underwent partial (n = 4) or total thyroidectomy (n = 82). Central (16 patients; 8 positive for metastases) and/or lateral (42 patients; 36 positive for metastases) lymph node dissections (LND) were performed in 58 patients. The tumor types included 31 classic PTC, 15 follicular variant, 5 tall cell, 5 columnar, 2 diffuse sclerosing, 1 cribriform morular, and 1 hobnail subtypes of PTC; 12 widely invasive follicular thyroid cancer (FTC); 4 oncocytic; and 10 poorly differentiated thyroid cancer (PDTC) (Fig. 1A). The median tumor size was 5 cm (range 0.7-13); 32 patients were in TNM stage 2 (young patients < 55 years with DM) and 41 patients were in TNM stage 4b (patients 55 years of age with DM) (Fig. 1B). The vast majority of patients were in the American Thyroid Association high-risk group (Fig. 1C).

**Table 1.** Clinical and pathological characteristics of 86 patients with thyroid cancer distant metastases

Characteristic	n
Sex (female:male) (%)	57:29 (66.3:33.7)
Median age (range), years	53.5 (10-82)
Surgery (%)	
Hemithyroidectomy	2 (2.3)
Bilateral partial thyroidectomy	2 (2.3)
Total thyroidectomy	82 (95.4)
LND (%)	
No LND	28 (32.6)
Berry picking	11 (12.8)
CND	6 (7.0)
Unilateral LND*^a^*	6 (7.0)
Bilateral LND*^a^*	6 (7.0)
CND and unilateral LND	16 (18.6)
CND and BND	13 (15.2)
Median tumor size (range) (cm)	5 (0.7-13)
Lymph node metastases (%)	58 (67.4)
Central	14 (16.3)
Lateral	11 (12.8)
Central and lateral	21 (24.4)
No lymph node metastases	12 (14)
No LND	28 (32.6)

Abbreviations: BND, bilateral lymph node dissection; CND, central lymph node dissection; LND, lymph node dissection.

^*a*^The standard lateral dissection in our patients was level II-IV. However, level V and VII are sometimes dissected if there are suspicious lymph nodes based on preoperative radiological evaluation.

### Pattern of DM

**Figure 2.**
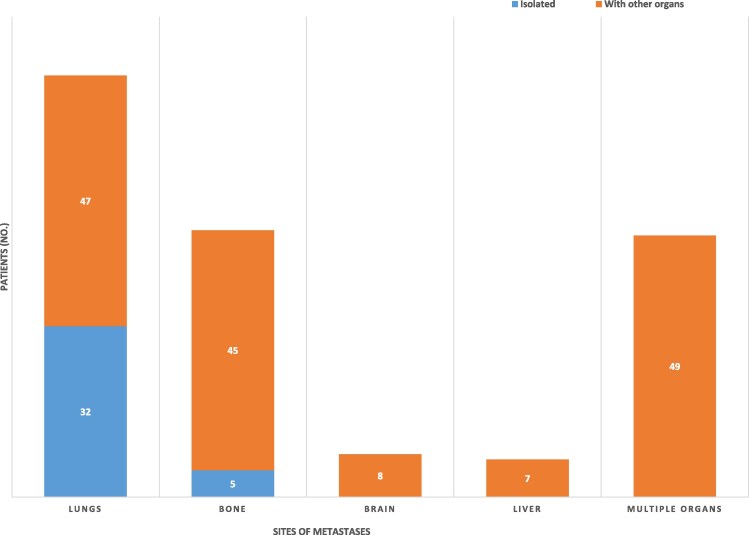
Bar chart showing the number of patients with different sites of thyroid cancer distant metastases occurring either isolated or in combination with metastases in other organs.

Seventy patients (81.4%) had synchronous DM at the time of diagnosis, while 16 (18.6%) developed DM during the follow-up (metachronous). Lung metastases were the most common, isolated in 32 patients (37.2%) or with DM in other organs in 47 patients (54.6%). Skeletal metastases occurred in 50 patients (58.1%): isolated in 5 patients (5.8%) and with DM in other organs in the other 45 patients (52.3%). Brain metastases occurred in 8 patients (9.3%), all with DM in other organs. Multiple-organ DM occurred in 49 patients (58%) (Fig. 2).

The predominant (heaviest burden) metastases involved the lungs in 43 patients (50%), the skeleton in 20 patients (23.3%), the lungs and bone in 12 patients (14%), as a locoregional disease in 4 patients (5%), and the lungs and brain in 3 patients (3.5%). Apart from the lungs, bone, and brain, the liver was the next most frequently involved organ, with liver metastases occurring in 7 patients (8%). Unusual sites of DM included subcutaneous abdominal fat, gluteus maximus muscle, ilium, adrenal gland, kidney, and esophagus. All of these DM occurred in the context of extensive disease in the more usual organs (lungs and bones).

The lung metastases were micro-metastases (< 1 cm) in 37 patients (46.8%) and macro-metastases (≥ 1 cm) in 42 patients (53.2%). F-18-fluorodeoxyglucose positron emission tomography/computed tomography scans were done in 64 patients (81%) with lung metastases and were positive in 47 (73.4%) of them (18 micro- and 29 macro-metastases) and negative in 17 of them (13 micro- and 4 macro-metastases). The most common sites for skeletal metastases were the spine, occurring in 41 patients (82%), alone in only 6 cases (12.0%) but with other sites of bone metastases in the other 35 patients (70%). Metastasis to the extremities, the ribs, and the skull occurred in 36 (41.8%), 20 (23.3%), and 16 (18.6%) patients, respectively. F-18-fluorodeoxyglucose positron emission tomography/computed tomography scans were done in 23 cases with skeletal metastases and were positive in 19 cases (82.6%).

### Management of Distant Metastases

All but 1 patient received 1 or more therapies for their DM, including locoregional disease when it occurred in combination with DM (Table 2). These included additional neck surgeries alone in 2 patients (2.3%), I-131 alone in 18 patients (20.9%), external beam radiotherapy alone in 5 patients (5.8%), and multikinase inhibitors alone in 2 patients (2.3%). The other 58 patients (67.4%) received 2 or more modalities of these therapies (range 2-6) (Table 2). Additional doses of I-131 were given to 73 patients (84.9%); the median cumulative I-131 administered activity was 346 mCi (range 49-1300). Multikinase inhibitors were given to 24 patients (28%) and were in the form of Sorafenib to 9 patients, Lenvatinib to 5 patients, Sorafenib followed by Lenvatinib in 10 patients.

**Table 2.** Additional therapies for distant metastases after the initial surgery and I-131 therapy

Intervention	n (%)
None	1 (1.2)
Surgery	2 (2.3)
I-131	18 (21)
XRT	5 (5.8)
TKI	2 (2.3)
Surgery + I-131	8 (9.3)
Surgery + XRT	3 (3.5)
I-131 + XRT	12 (14)
I-131 +TKI	1 (1.2)
Surgery + I-131 + XRT	13 (15.1)
Surgery + I-131 + TKI	3 (3.5)
I-131 + XRT + TKI	8 (9.3)
Surgery + I-131 + XRT + TKI	10 (11.6)
Total	86 (100)

Abbreviations: I-131, radioactive iodine; TKI, tyrosine kinase inhibitor; XRT, external beam radiotherapy.

### Outcome of Distant Metastasis

The median follow-up for the whole cohort was 84 months (IQR 35.5-118). During this period, 47 patients succumbed to their disease (ie, disease-specific mortality 54.7%). The median time from diagnosis of TC to death was 47 months (IQR 16-84). In bivariate analyses, factors associated with mortality were increasing age (*P* = .001) and bone metastases (*P* < .0001) (Table 3). These factors remained significant in a multivariate analysis (Table 3). Although 7 out of 8 patients with brain metastases died and only 1 remained alive, this did not reach statistical significance, most likely due to the small number of patients with brain metastases. Sex, time of appearance of DM (synchronous or metachronous), and use of tyrosine kinase inhibitors (used in 25 patients) did not predict mortality. Similarly, *BRAF*^V600E^, *TERT* promoter mutations, or both did not predict mortality, but this might be due to the small number of samples tested (41 patients) (Table 3).

**Table 3.** Univariate and multivariate analyses of predictive factors of cancer-specific mortality comparing patients with thyroid cancer metastases who are still alive with those who died

			*P*-value	
Characteristic	Alive (n =39)	Died (n = 47)	Unadjusted	Adjusted*^a^*
Median age (range)	49 (29-60)	59 (51-59)	.001	.009
Age (≥ 50 years)*^b^*	17 (43.6)	39 (83.0)	<.0001	.027
Sex (female:male)	27:12	30:17	.76	
Distant metastases (at the time of initial diagnosis)	29 (41.4)	41 (51.6)	.21	
Lung metastases	36 (92.3)	43 (91.5)	1.0	
Bone metastases	13 (33.3)	37 (78.7)	<.0001	.017
Brain metastases	1 (2.6)	7/47 (14.9)	.067	
Use of TKIs	10 (25.6)	15 (31.9)	.69	
*BRAF* ^V600E^ mutation	7/19 (36.8)	7/22 (31.8)	.75	
*TERT* promoter mutations	7/19 (36.8)	14/22 (63.3)	.12	
*BRAF* ^V600E^/*TERT* promoter mutations	11/19 (57.9)	16/21 (76)	.19	

Abbreviation: TKI, tyrosine kinase inhibitor.

^*a*^Using Cox proportional hazards ratio analysis.

^*b*^Using receiver operating characteristic curve to determine the best cutoff value for age in predicting mortality, age 50 years was the best value with area under the curve 0.703 (95% confidence interval 0.59-0.82), *P* .001, sensitivity 0.83, specificity 0.56.

### Survival Analyses

**Figure 3.**
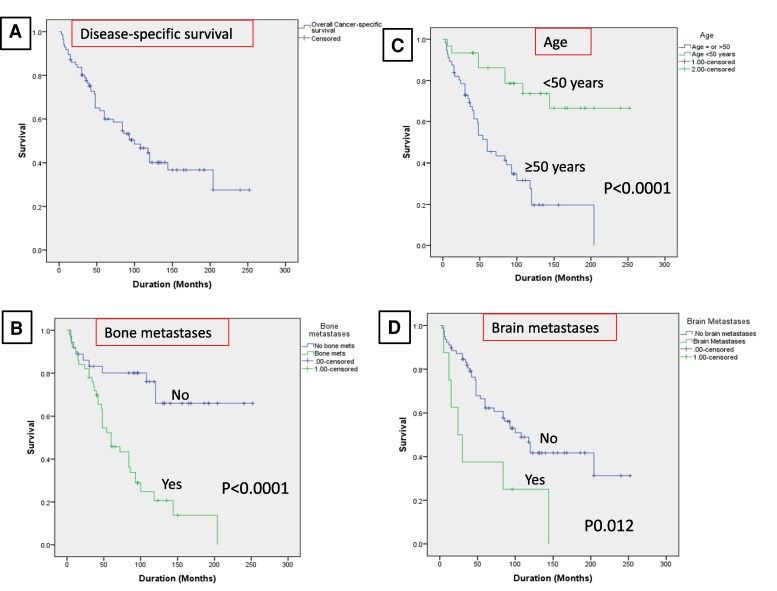
Kaplan-Meir analysis showing overall disease-specific survival in the whole cohort of 86 patients with thyroid cancer distant metastases (A), in patients with and without bone metastases (B), by age (C), and in patients with and without brain metastases (D).

In a Kaplan-Meier analysis (Fig. 3), increasing age, bone metastases, and brain metastases were significantly associated with survival. However, in a multivariate analysis using the Cox proportional hazards model in which cancer-specific death was the dependent variable and increasing age, bone metastases, and brain metastases were the covariates, only increasing age and bone metastases remained significant (Table 3). The median survival for those who were still alive was 108 months (95% confidence interval 90-150) and only 47 months (IQR 16-84) for those who died.

## Discussion

DM are the cause of significant morbidity and the major cause of death in DTC [9, 11, 14]. In this study, the lungs were the most common site of metastases occurring in approximately 92% of this cohort and were the sole site in 32 patients (37%). However, except for bone metastases, which occurred in 5 patients as the sole sites of metastases, all other patients (57%) had multiorgan disease. Unlike bone metastases, which predominantly affected those who died, lung metastases were common in patients who died and those who are still alive, and this explains the lack of association between lung metastases and mortality. Lung metastases were found in some studies to be the main predictor of mortality [14, 15], while other studies did not find such a relationship [16].

We found that DM are associated with high mortality despite the considerable advances in the management of DTC in general and metastatic disease in particular, including the use of multikinase inhibitors for more than a decade. The DM-associated mortality in this cohort was approximately 55% over a median survival time of about 4 years. Age and skeletal metastases were the major determinants of mortality. Although most patients with brain metastases died (7/8), the number of patients was too small to maintain significance in a multivariate analysis.

In many previous studies, age has been found to affect the outcome and risk of mortality [14, 17-22] and is an integral factor of the TNM staging system and almost all previous staging systems [23, 24]. In a recent large study that analyzed the outcome of >2000 patients with DM of thyroid cancer using the Surveillance, Epidemiology, and End Results database, age was a strong predictive factor of mortality [14]. In that study, increasing age was a significant factor when used as a binary factor, with an age cutoff limit of ≥ 58 years being a strong predictor of disease-specific survival and overall survival [14]. Increasing age was also an important predictive factor of overall survival and disease-specific survival when used as a continuous variable and, in combination with other risk factors, provided a more granular risk estimate of mortality [14]. Similarly, our study shows increasing age as a strong predictive factor, whether analyzed as a continuous or a dichotomous variable. Using receiver operating characteristic curve analysis, we found that an age of 50 years was the best cutoff age for predicting disease-specific mortality, and both direct and survival bivariate and multivariate analyses showed a consistent predictive effect of age on the risk of mortality in metastatic DTC. In another study, age > 55 years was also found to be associated with less likelihood of achieving an excellent response with less response to therapy and an increased risk of death in those who had an incomplete response [25]. The reason for this age-related worse outcomes is not fully understood, but studies have shown less expression of sodium iodide symporter (NIS) on DTC from old patients, and NIS is a major determinant of response to I-131 [26, 27]. In addition, the incidence of *BRAF*^V600E^ and *TERT* promoter mutations, 2 of the most important molecular markers of aggressiveness of DTC, is higher in DTC of patients with increasing age [28-30]. A study has shown that age is a strong prognostic factor in DTC with *BRAF*^V600E^ and not in the wild-type *BRAF* DTC [28, 31, 32].

Bone metastases are the other factors that were significantly associated with mortality in this cohort. Bone metastases are rare, occurring in about 4% of all patients with DTC, but are associated with significant morbidity and mortality [5, 33-36]. This is even worse in patients with PDTC, who have a 10-year overall survival of only 15% [33, 37]. Bone metastases are more likely to occur with FTC and PDTC [37]. They are usually I-131 refractory, and although generally responsive to tyrosine kinase inhibitors, their response is less than lung metastases [38]. Local measures such as surgery, external beam radiotherapy, stereotactic radiotherapy, thermal ablation, and arterial embolization have been used in the management of bone metastases [33]. Antiresorptive drugs such as bisphosphonate and denosumab might have an antineoplastic and antiresorptive effect by working on the tumor microenvironment [39]. In our cohort, bone metastases were common, probably due to the high rates of the types of thyroid cancer that have a high propensity for skeletal metastases such as FTC and PDTC. The high proportion of patients with bone metastases likely explains the higher mortality in our cohort than in many other cohorts with DM. In a recent study of 82 patients with bone metastases, age, tumor size, and the presence of extraosseous metastases were factors associated with mortality. These findings were further confirmed by analyzing 287 DTC patients from the Surveillance, Epidemiology, and End Results database [40]. These factors are similar to factors found in our patients except that tumor size was not a significant factor in our study, probably due to the dominating effect of the DM and lack of data on tumor size in some patients.

None of the usual risk factors for DM, such as tumor size or lymph node metastases, was significantly associated with cancer-specific mortality in this study. This is understandable, as these factors increase the risk of DM. However, once metastases are established, these factors become less important, and the metastases themselves predominate as determinants of mortality. Although *BRAF*^V600E^ and *TERT* promoter mutations have been reported to be associated with more aggressive disease and mortality risks, in this study, they were not associated with disease-specific mortality. Although there seems to be a signal of association between the *TERT* promoter or the combination of *BRAF*^V600E^ and *TERT* promoter mutations, this did not reach statistical significance. However, less than 50% of patients in this cohort had these mutations tested, and this may be the reason for the lack of association between these mutations and cancer-specific mortality.

This study has some strengths and some limitations. It presents data from a tertiary care center with extensive experience in managing thyroid cancer with more than 7000 patients treated over the last 3 decades. The data also are from an infrequently studied population. The standard of care follows international guidelines, and the treating physicians are all North American-trained. However, the number of patients studied is limited (86 patients), and there might be selection bias related to the nature of tumor registries from which this cohort was derived. DM with a limited number or impact may not have been registered. This is also suggested by the high mortality and the high percentage of multiorgan disease. Nevertheless, the data is extensively analyzed and adds knowledge to the rare and not well-studied DM, especially those in unusual sites such as the bone, brain, and liver.

In summary, DM are a major cause of mortality in patients with DTC, leading to death in more than 50% within a median time of about 4 years. Bone metastases in particular are associated with very high mortality, and increasing age is a major determinant of death in patients with DM.

## Data Availability

Original data generated and analyzed during this study are included in this published article.
